# Glucagon-like peptide-1 receptor agonists for heart failure with reduced ejection fraction: Is it the next step?

**DOI:** 10.1093/eschf/xvag131

**Published:** 2026-05-07

**Authors:** Jan Biegus

**Affiliations:** Department of Cardiology, Clinical Department of Intensive Cardiac Care, Faculty of Medicine, Wroclaw Medical University, Institute of Heart Diseases, Borowska 213, Wroclaw 50-556, Poland; Department of Cardiology, Jan Mikulicz Radecki University Hospital in Wrocław, Borowska 213, Wroclaw 50-556, Poland

**Keywords:** GLP-1 agonist, HFrEF, Therapy

The adipokine hypothesis has emerged as a novel framework to explain the pathophysiology of heart failure with preserved ejection fraction (HFpEF).^1^ In this paradigm, visceral adipose tissue is no longer viewed as a passive energy reservoir but rather as a metabolically active, endocrine organ that secretes a broad array of bioactive mediators called adipokines that exert systemic and local cardiovascular effects.^1^ These molecules promote key pathobiological processes central to HFpEF, including myocardial fibrosis, endothelial dysfunction, microvascular maladaptation, and chronic low-grade inflammation, thereby shaping the syndrome's characteristic clinical phenotype.^2,3^ Visceral adipose tissue is considered a principal source of these maladaptive signals and therefore a pathophysiological cause of the syndrome.

This conceptual shift carries important therapeutic implications. If dysfunctional adipose tissue is a driver of disease, it becomes a rational target for intervention.^4–7^ In this context, glucagon-like peptide-1 receptor agonists (GLP-1a), which induce reductions in adipose tissue mass and favourably modulate metabolic and inflammatory pathways, have garnered increasing attention as therapies for HFpEF.^8^ Early clinical studies in HFpEF populations demonstrated that GLP-1a may improve functional capacity, reduce weight, and enhance patient-reported outcomes, decrease pulmonary artery pressure, supporting the notion that targeting adipose biology may translate into clinically meaningful benefits.^9–11^

However, this framework also raises a critical question that has been insufficiently addressed. If adipose tissue exerts broadly deleterious cardiovascular effects, and if excess adiposity is highly prevalent across the entire spectrum of HF irrespective of ejection fraction, why should its impact be considered specific to HFpEF only? In other words, does adipose-driven pathophysiology also contribute meaningfully to heart failure with reduced ejection fraction (HFrEF), and if so, may the GLP1a provide some benefit in HFrEF as well?

Notably, emerging data suggest that the biological effects of GLP-1a extend beyond weight reduction and may confer non-phenotype-specific cardiovascular benefits that would be expected to benefit patients across the ejection fraction spectrum. This raises the possibility that the therapeutic relevance of targeting adipose tissue—and adipokine signalling—may transcend the traditional HFpEF-centric narrative.

Re-evaluating the adipokine hypothesis from a broader, ejection fraction-independent perspective may be crucial to enhancing our understanding of HF biology and better integrating new metabolic therapies into the evolving treatment landscape.

At present, there is no evidence of benefit supporting GLP-1 receptor agonists as HF–specific therapy in patients with HFrEF and obesity. Although earlier trials with liraglutide evaluated patients with HFrEF, they did not demonstrate clinical benefit and raised safety concerns.^12,13^ GLP-1a may increase heart rate, risk of arrhythmias, and were reported to be associated with a higher risk of HF hospitalizations.^14^ In the SELECT trial, which recruited patients with BMI ≥27 kg/m^2^ and established cardiovascular disease, 1347 patients had diagnosed HFrEF. Importantly, there was no evidence of harm from GLP-1a in this cohort, but these data are based on a subgroup analysis. Given that, GLP-1 receptor agonists should not currently be positioned as disease-modifying HFrEF therapy. Rather, in patients with HFrEF and obesity, their use may be considered primarily as obesity treatment, with potential downstream cardiometabolic benefits, but without dedicated evidence for improving HFrEF outcomes.

Since GLP-1 receptor agonists are approved for obesity treatment and are not formally contraindicated in HFrEF, their use in this context is clinically plausible. In this issue of ESC Heart Failure, Kassab et al. have provided real-world data on GLP-1a use in HFrEF (mean age ∼62 years, mean EF∼30 ± 8.7%) and its associations with prognosis. As this was a retrospective analysis, the authors performed propensity score matching to adjust for some obvious differences between patients on and off the therapy.^15^ After adjustments, the GLP-1a group had a significantly lower risk of the primary outcome (1-year all-cause mortality and HF hospitalizations), with an OR (95% CI): 0.68 (0.51–0.90). Importantly, GLP-1a use was associated with improvements in both components of the composite endpoint separately.^15^ Moreover, there was also no signal of potential harm in terms of other cardiovascular events, such as acute coronary syndrome, stroke, or arrhythmias.

We can only speculate that the full spectrum of biological effects (benefits) of the anti-obesity drugs may be observed in patients with a greater burden on adipose tissue. Indeed, the mean BMI in patients recruited to the clinical trials was 29 kg/m^2^ (LIVE), 32 kg/m^2^ (FIGHT), and 33 kg/m^2^ (SELECT), while in the real-world population reported by Kassab et al., the mean BMI was higher: 34.9%. Thus, if this hypothesis is true, the degree of weight loss should be clinically significant and associated with outcomes.

Although this data appears promising, it should be considered a hypothesis-generating signal due to the study's clear limitations. Nevertheless, the authors deserve praise for their work and for offering valuable clinical insights, especially regarding safety. Of course, no final conclusions can be drawn from this study, and only future randomized controlled trials (RCTs) in HFrEF can offer definitive answers.

## Declarations

### Data Availability

No new data were generated or analyzed in support of this editorial. All data discussed are derived from previously published studies.

## References

[1] Packer M . The adipokine hypothesis of heart failure with a preserved ejection fraction. J Am Coll Cardiol 2025;86:1269–373. 10.1016/j.jacc.2025.06.055 40886173 PMC12766646

[2] Lembo M, Strisciuglio T, Fonderico C, Mancusi C, Izzo R, Trimarco V, et al Obesity: the perfect storm for heart failure. ESC Heart Fail 2024;11:1841–60. 10.1002/ehf2.14641 38491741 PMC11287355

[3] Crum Y, Hoendermis ES, van Veldhuisen DJ, van Woerden G, Lobeek M, Dickinson MG, et al Epicardial adipose tissue and pericardial constraint in heart failure with preserved ejection fraction. ESC Heart Fail 2024;11:1698–706. 10.1002/ehf2.14739 38438270 PMC11098664

[4] Butler J, Shah SJ, Magwire M, Campos C, Usman MS, Hoovler A, et al Treatment pathways in patients with heart failure with preserved ejection fraction and obesity: perspectives from cardiology specialists and patients. Global Cardiol 2024;2:108–16. 10.4081/cardio.2024.38

[5] Anker SD, Ji L, Kindel T, Coats AJS, Ojji D, Puente Barragán A, et al iCARDIO alliance global implementation guidelines for the management of obesity 2025—focus on prevention and treatment of cardiometabolic disease. Global Cardiol 2025;3:181–206. 10.4081/cardio.2025.86 PMC13261179 42291066

[6] Cimino G, Vaduganathan M, Lombardi CM, Pagnesi M, Vizzardi E, Tomasoni D, et al Obesity, heart failure with preserved ejection fraction, and the role of glucagon-like peptide-1 receptor agonists. ESC Heart Fail 2024;11:649–61. 10.1002/ehf2.14560 38093506 PMC10966224

[7] Rosano GMC, Coats AJS. Modulation of cardiac metabolism in heart failure. Global Cardiol 2024;2:133–8. 10.4081/cardio.2024.30

[8] Berger M, Marz W, Niessner A, Delgado G, Kleber M, Scharnagl H, et al IL-6 and hsCRP predict cardiovascular mortality in patients with heart failure with preserved ejection fraction. ESC Heart Fail 2024;11:3607–15. 10.1002/ehf2.14959 39003598 PMC11631318

[9] Jiang H, Wattanachayakul P, Kittipibul V, Nicolsen E, McVeigh T, Kamneva O, et al Pulmonary artery pressure trajectories in heart failure patients treated with GLP-1 receptor agonists. ESC Heart Fail 2025;12:2578–82. 10.1002/ehf2.15308 40289068 PMC12287838

[10] Packer M, Zile MR, Kramer CM, Baum SJ, Litwin SE, Menon V, et al Tirzepatide for heart failure with preserved ejection fraction and obesity. N Engl J Med 2025;392:427–37. 10.1056/NEJMoa2410027 39555826

[11] Kosiborod MN, Abildstrøm SZ, Borlaug BA, Butler J, Rasmussen S, Davies M, et al Semaglutide in patients with heart failure with preserved ejection fraction and obesity. N Engl J Med 2023;389:1069–84. 10.1056/NEJMoa2306963 37622681

[12] Margulies KB, Hernandez AF, Redfield MM, Givertz MM, Oliveira GH, Cole R, et al Effects of liraglutide on clinical stability among patients with advanced heart failure and reduced ejection fraction: a randomized clinical trial. JAMA 2016;316:500–8. 10.1001/jama.2016.10260 27483064 PMC5021525

[13] Jorsal A, Kistorp C, Holmager P, Tougaard RS, Nielsen R, Hänselmann A, et al Effect of liraglutide, a glucagon-like peptide-1 analogue, on left ventricular function in stable chronic heart failure patients with and without diabetes (LIVE)—a multicentre, double-blind, randomised, placebo-controlled trial. Eur J Heart Fail 2017;19:69–77. 10.1002/ejhf.657 27790809

[14] Marques P, Travlos CK, Matias P, Neves JS, Tsoukas MA, Mavrakanas TA, et al Effects of glucagon-like peptide 1 receptor agonist initiation in patients with heart failure with reduced ejection fraction and implantable cardiac devices. JACC Heart Fail 2025;13:102573. 10.1016/j.jchf.2025.102573 40992090

[15] Kassab J, Drazner MH, Thibodeau JT. (April 17, 2026) Glucagon-like peptide-1 receptor agonists in patients with heart failure with reduced ejection fraction. ESC Heart Fail, 10.1093/eschf/xvag111 PMC13220942 41991158

